# Teicoplanin – Tigecycline Combination Shows Synergy Against *Mycobacterium abscessus*

**DOI:** 10.3389/fmicb.2018.00932

**Published:** 2018-05-11

**Authors:** Dinah B. Aziz, Jeanette W. P. Teo, Véronique Dartois, Thomas Dick

**Affiliations:** ^1^Department of Medicine, Yong Loo Lin School of Medicine, National University of Singapore, Singapore, Singapore; ^2^Department of Pharmacy, Faculty of Science, National University of Singapore, Singapore, Singapore; ^3^Department of Laboratory Medicine, National University Hospital, Singapore, Singapore; ^4^The Public Health Research Institute, Rutgers, New Jersey Medical School, The State University of New Jersey, Newark, NJ, United States; ^5^Department of Microbiology and Immunology, Yong Loo Lin School of Medicine, National University of Singapore, Singapore, Singapore

**Keywords:** *Mycobacterium abscessus*, teicoplanin, tigecycline, synergy, repurposing

## Abstract

Lung disease caused by non-tuberculous mycobacteria (NTM), relatives of *Mycobacterium tuberculosis*, is increasing. *M. abscessus* is the most prevalent rapid growing NTM. This environmental pathogen is intrinsically resistant to most commonly used antibiotics, including anti-tuberculosis drugs. Current therapies take years to achieve cure, if cure if achieved. Thus, there is an urgent medical need to identify new, more efficacious treatments. Here, we explore the possibility of repurposing antibiotics developed for other indications. We asked whether novel two-drug combinations of clinically used antibiotics can be identified that show synergistic activity against this mycobacterium. An *in vitro* checkerboard titration assay was employed to test 180 dual combinations of 41 drugs against the clinical isolate *M. abscessus* Bamboo. The most attractive novel combination was further profiled against reference strains representing three sub-species (*M. abscessus* subsp. *abscessus*, *massiliense* and *bolletii*) and a collection of clinical isolates. This resulted in the identification of a novel synergistic antibiotic pair active against the *M. abscessus* complex: the glycopeptide teicoplanin with the glycylcycline tigecycline showed inhibitory activity at 2–3 μM (teicoplanin) and 1–2 μM (tigecycline). This novel combination can now be tested in *M. abscessus* animal models of infection and/or patients.

## Introduction

Among the rapid growing non-tuberculous mycobacteria (NTM), *M. abscessus* is the most common cause of lung disease (Griffith et al., 2007; Medjahed et al., 2010; Hoefsloot et al., 2013). A poor rate of successful chemotherapeutic treatment makes *M. abscessus* disease a chronic incurable infection (Griffith et al., 2007). The bacterium is intrinsically drug resistant to most antibiotics (Brown-Elliott et al., 2012; Nessar et al., 2012). Currently, *M. abscessus* infections are treated by a multi-drug regimen consisting of a macrolide (clarithromycin), amikacin and either cefoxitin or imipenem (Benwill and Wallace, 2014; Ryu et al., 2016). Different clinics may choose to add on additional antibiotics and recently, tigecycline has been used (Wallace et al., 2014; Floto et al., 2016). The treatment issues are further complicated by the ability of two out of three sub-species of *M. abscessus* to develop macrolide resistance upon exposure to sub-inhibitory concentrations of the drug (Nash et al., 2009; Bastian et al., 2011; Maurer et al., 2014). Indeed, a recent study conducted in a hollow fiber model showed that the standard regimen of clarithromycin, amikacin, and cefoxitin exerted low sterilizing activity within the first 14 days of treatment, and re-growth of the bacteria was seen after this period due to inducible macrolide resistance (Ferro et al., 2016). Demonstrated transmission of *M. abscessus* between cystic fibrosis patients (Bryant et al., 2016) has increased the urgency to identify novel treatments for this NTM pathogen.

Screening for synergy interactions of approved drugs is an approach to new medicines that allows rapid bench-to-bedside translation (Hill and Cowen, 2015). A series of synergy studies have been conducted for *M. abscessus* and among the combinations that have been identified so far are imipenem + clarithromycin, imipenem + levofloxacin, clarithromycin + linezolid, clarithromycin + vancomycin, clofazimine + amikacin, tigecycline + clarithromycin, tigecycline + clofazimine, tigecycline + linezolid, clavulanate + meropenem, doripenem + rifampicin, biapenem + rifampicin, avibactam + ertapenem, avibactam + tebipenem, and avibactam + panipenem (Miyasaka et al., 2007; Cremades et al., 2009; Shen et al., 2010; van Ingen et al., 2012; Huang et al., 2013; Oh et al., 2014; Singh et al., 2014; Kaushik et al., 2015, 2017; Mukherjee et al., 2017).

To identify novel synergistic combinations, we carried out a large scale study using the checkerboard assay employing two different strategies. The first strategy was to screen combinations of β-lactams with β-lactamase inhibitors (Livermore, 1995; Bebrone et al., 2010). *M. abscessus* harbors the *bla*_mab_ gene encoding an Ambler class A β-lactamase (Soroka et al., 2014) and an inhibitor might restore activity of β-lactams against *M. abscessus*. The second strategy was to screen combinations of cell wall-targeting antibiotics with antibiotics that engage intracellular targets. This approach is based on our previous findings that vancomycin displayed (moderate) activity against *M. abscessus* (Aziz et al., 2017) and showed synergy with clarithromycin (Mukherjee et al., 2017). We screened a total of 180 dual drug combinations against a clinical isolate of *M. abscessus* and found that the combination of teicoplanin and tigecycline displayed synergistic activity. We characterized the *in vitro* activity of this novel combination against *M. abscessus* reference strains and diverse clinical isolates.

## Materials and Methods

### Compounds

The 36 antibiotics and 5 β-lactamase inhibitors used in this study were obtained from commercial sources and dissolved according to the manufacturer’s recommendations. Teicoplanin was obtained from Sigma-Aldrich, while tigecycline was obtained from Adooq BioScience. Both antibiotics were dissolved in 90% dimethyl sulfoxide (DMSO).

### Bacterial Strains and Culture Media

*Mycobacterium abscessus* Bamboo (Yee et al., 2017) was used for screening of combinations and the subsequent confirmation of synergy hit combinations. For the checkerboard titration assay determination of the activity of the teicoplanin + tigecycline hit against the various *M. abscessus* subspecies within the *M. abscessus* complex, *M. abscessus* subsp. *abscessus* (ATCC 19977), *M. abscessus* subsp. *bolletii* (CCUG 50184-T) and *M. abscessus* subsp. *massiliense* (CCUG 48898-T) were used. Reference strains were obtained from the American Type Culture Collection (ATCC) and the Culture Collection University of Goteborg (CCUG), respectively. For further characterization of the teicoplanin + tigecycline combination in the macrolide resistance induction assay, *M. abscessus* subsp. *abscessus* (ATCC 19977) harboring the T28 sequevar of *erm41* gene, conferring inducible resistance upon exposure to sub-inhibitory concentrations of macrolides (Nash et al., 2009; Bastian et al., 2011) was used. For determination of synergy of teicoplanin + tigecycline against a variety of clinical isolates, strains were obtained from the strain collection of the clinical microbiology laboratory at the National University Hospital, Singapore. The strains were characterized by the lab as previously described (Aziz et al., 2017). For the evaluation of the bactericidal activity of the synergy combination *M. abscessus* subsp. *abscessus* (ATCC 19977) was used.

Liquid cultures were grown in standard mycobacterium medium, Middlebrook 7H9 broth (BD Difco) supplemented with 0.5% albumin, 0.2% glucose, 0.085% sodium chloride, 0.0003% catalase, 0.2% glycerol and 0.05% Tween 80. Solid cultures were grown on Middlebrook 7H10 agar (BD Difco) supplemented with 0.5% albumin, 0.2% glucose, 0.085% sodium chloride, 0.5% glycerol, 0.0003% catalase and 0.006% oleic acid.

*Mycobacterium abscessus* bacterial work was carried out under BSL-2 conditions according to approved biosafety protocols.

### Checkerboard Titration Assay

This assay was carried out in 96-well microtiter plates as previously described (Hsieh et al., 1993; Kaushik et al., 2015), with some modifications. Drugs were tested within the range of concentrations of either 0–25 μM or 0–50 μM, at twofold serial dilutions. For each combination, 8 concentrations of a drug were tested for synergy against 11 concentrations of another drug. Hence, for each two-drug combination screened for synergy, 88 different combination concentrations are tested. A total of 180 different two-drug combinations were tested in this study. For the screening of combinations, this assay was carried out using the Tecan D300e Digital Dispenser for dispensing of drugs. For confirmation of the teicoplanin + tigecycline hit, as well as its subsequent characterization against sub-species, clinical isolates and induced cultures, drugs were dispensed manually. Results were reproducible between the two methods of dispensing drugs for this assay. Briefly, this assay was carried out in 96-well flat bottom plates, with two different compounds, with a starting inoculum of an optical density at 600 nm (OD_600_) of 0.05 (10^7^ colony forming units or cfu/mL) in a final volume of 200 μL. The culture for the starting inoculum was diluted from a pre-culture at mid-log phase (OD_600_ = 0.4 to 0.6). The plates were sealed using parafilm, put in an airtight container with moist tissue and incubated for 3 days at 37°C on an orbital shaker at 110 rpm. Each plate had a media-only control, a drug free control as well as a positive control of clarithromycin at 20 μM. After 3 days of incubation, the cultures in the wells were manually re-suspended before OD_600_ was read in the plate reader (Tecan Infinite 200 Pro) and used to calculate growth inhibition percentage of each well. The Fractional Inhibitory Concentration Index (FICI) was used to analyze the results from the checkerboard assay. FICI was calculated by using the concentrations at which at least 90% inhibition of the culture in the well as compared to the drug free culture was observed. It was computed as FICI = [(concentration of drug A in combination/concentration of drug A when used alone) + (concentration of drug B in combination/concentration of drug B when used alone)] (Hsieh et al., 1993). Synergy is defined as FICI ≤ 0.5, indifference is defined as 0.5 < FICI ≤ 4, and antagonism is defined as FICI > 4 (Hsieh et al., 1993).

### Macrolide Resistance Induction Assay

*M. abscessus* subsp. *abscessus* (ATCC 19977) mid-log phase culture was diluted to OD_600_ = 0.05 and treated with clarithromycin at a sub-inhibitory concentration of 0.075 μM (fourfold lower than clarithromycin MIC_50_ (concentration that causes 50% growth inhibition). An untreated culture was set up as a control. Cultures were grown to mid-log phase overnight and then subjected to the checkerboard titration assay as described above.

### Bactericidal Assay

Bactericidal activity determinations were carried out in 14 mL round bottom tubes with the compounds added at set concentrations, with a starting inoculum of OD_600_ 0.05 (10^7^ cfu/mL) in a final volume of 1 mL. The culture for the starting inoculum was diluted from a pre-culture at mid-log phase (OD_600_ = 0.4 to 0.6). Tubes were incubated for 3 days at 37°C with shaking at 160 rpm. After 3 days of drug exposure, 10 μL of the cultures were plated at different dilutions in 12 well plates containing 2 mL of 7H10 agar in each well. The plates were sealed with parafilm and incubated at 37°C for 4 days and then colonies were counted. We report fold-kill, which is the reduction in cfu/mL of the treated culture compared to the time zero untreated control.

## Results

### Screening of 180 Two-Drug Combinations for Synergy Against *M. abscessus* Identifies 11 Hits

We screened a total of 180 two-drug combinations of approved antibiotics for their growth inhibition potency against the clinical isolate *M. abscessus* Bamboo using the checkerboard assay. Hits were defined as combinations that showed at least a fourfold decrease in concentration of each drug that was needed to achieve 90% inhibition as compared to the concentration needed to achieve that same level of inhibition when either drug was used alone. Screening of β-lactam – β-lactamase inhibitor combinations, identified 6 hits out of 110 combinations (**Table 1**). Screening of combinations of cell envelope targeting drugs with antibiotics that inhibit intracellular targets, identified 5 hits out of 70 combinations (**Table 2**). Taken together, the screen identified 11 primary hits (6.1% hit rate) which were re-confirmed with fresh solids (**Table 3**).

**Table 1:** Outcome of screening 110 combinations of β-lactams and β-lactamase inhibitors against *Mycobacterium abscessus* Bamboo: 6 two-drug hits.

		β-lactamase inhibitors					
		
		Non-β-lactam- based			β-lactam-based		
			
			AVI	VAB	CLA	SUL	TZB
β-lactams	Carbapenems	Biapenem	I	I	N.D	N.D	N.D
		Doripenem	I	I	I	I	I
		Ertapenem	I	I	N.D	N.D	N.D
		Faropenem	I	N.D	I	I	I
		Imipenem	I	I	I	I	I
		Meropenem	I	I	I	I	I
		Panipenem	S	S	I	I	I
		Tebipenem	S	S	N.D	N.D	N.D
	Cephalosporins	Cefaclor	I	I	I	I	I
		Cefprozil	I	N.D	I	I	I
		Cefoxitin	I	I	I	I	I
		Cefdinir	I	N.D	I	I	I
		Cefditoren	I	I	I	I	I
		Cefixime	I	I	I	I	I
		Ceftiofur	I	I	I	I	I
		Cefoperazone	I	I	I	I	I
		Ceftazidime	I	I	I	I	I
		Cefozopran	I	N.D	I	I	I
		Ceftobiprole	I	N.D	I	I	I
	Penicillins	Ampicillin	S	I	I	I	I
		Amoxicillin	S	I	I	I	I
		Cloxacillin	I	I	I	I	I
		Methicillin	I	N.D	I	I	I
		Piperacillin	I	I	I	I	I
		Ticarcillin	I	I	I	I	I


*S, synergistic; I, indifferent; N.D, not determined. Synergy is defined as FICI ≤ 0.5. However, FICI values cannot be calculated for combinations involving β-lactamase inhibitors or other drugs that do not have MIC values when used individually. Hence, in the tables, synergistic interactions are defined as either combinations with FICI ≤ 0.5 or combinations that exhibit potentiation where a fourfold or more reduction in concentration of both drugs when used together is observed to inhibit 90% of growth as compared to when they are each used alone. Indifferent is defined as 0.5 < FICI ≤ 4. Where FICI cannot be calculated, ‘indifferent’ is defined as a less than fourfold in concentration of each antibiotic needed to achieve inhibition of 90% of growth when used together as compared to when the antibiotic is used alone. AVI, avibactam; VAB, vaborbactam; CLA, clavulanate; SUL, sulbactam; TZB, tazobactam.*

**Table 2:** Outcome of screening 70 combinations of cell envelope-targeting antibiotics with antibiotics targeting intracellular targets against *M. abscessus* Bamboo: 5 synergistic two-drug hits.

			Antibiotics with intracellular targets					
			
			LZD	LVX	MXF	AZM	CLR	TGC	RFB
Cell envelope-targeting antibiotics	β-lactams	Doripenem	I	I	I	I	I	N.D	I
		Faropenem	I	I	I	I	I	N.D	I
		Imipenem	I	N.D	I	I	I	N.D	I
		Panipenem	I	I	I	I	I	N.D	I
		Cefoxitin	I	I	I	I	I	N.D	I
		Cefdinir	I	I	I	I	I	N.D	I
		Cefozopran	I	I	I	I	I	N.D	I
		Ceftiofur	I	I	I	I	I	N.D	I
		Ceftobiprole	S	I	I	I	I	N.D	I
	Glycopeptides	Ramoplanin	I	I	I	N.D	S	S	I
		Vancomycin	N.D	N.D	N.D	N.D	S^a^	S	I
		Teicoplanin	I	I	I	I	I	S	I
	Polymyxins	Colistin	N.D	I	I	N.D	N.D	N.D	N.D


*S, synergistic; I, indifferent; N.D, not determined. For definition of terms see legend of **Table 1**. LZD, linezolid; LVX, levofloxacin; MXF, moxifloxacin; AZM, azithromycin; CLR, clarithromycin; TGC, tigecycline; RFB, rifabutin. ^*a*^Reported previously in Mukherjee et al. (2017).*

**Table 3:** Reconfirmation of 11 two-drug hits identified from screening of 180 combinations against *M. abscessus* Bamboo.

Strategy	Combination	MIC_90_ (μM)	
		
		Alone	Combined
β-lactams + β-lactamase inhibitors	Panipenem +	>50	50


	Avibactam	>50	6
	Tebipenem +	46	13
	Avibactam	>50	0.8
	Ampicillin +	>50	25
	Avibactam	>50	19
	Amoxicillin +	>50	25
	Avibactam	>50	25
	Panipenem +	>50	50
	Vaborbactam	>50	2
	Tebipenem +	40	10
	Vaborbactam	>50	10
Cell envelope-targeting + antibiotics with intracellular targets	Ceftobiprole +	>50	38


	Linezolid	>50	19
	Ramoplanin +	30	5
	Clarithromycin	0.7	0.2
	Ramoplanin +	30	5
	Tigecycline	7	0.8
	Vancomycin +	17	2
	Tigecycline	6	1
	Teicoplanin +	25	3
	Tigecycline	8	1


*The synergy or potentiation concentration of the 11 drug pair hits identified in the screens shown in **Tables 1**, **2** are reported. Results shown are the mean of two replicates. Standard deviations were ±50% of shown values.*

Three of our combination hits, panipenem + avibactam, tebipenem + avibactam, and amoxicillin + avibactam were reported previously (Dubee et al., 2015a; Kaushik et al., 2017).

Out of our eight novel hits, the potencies of ampicillin + avibactam, panipenem + vaborbactam, tebipenem + vaborbactam, and ceftobiprole + linezolid were only modest, with MIC_90_ (concentrations that inhibit 90% of growth) of 25 + 19 μM, 50 + 2 μM, 10 + 10 μM, and 38 + 19 μM (**Table 3**).

One of our novel hits involved the glycopeptide ramoplanin in combination with clarithromycin, however, this is not unexpected since we had previously reported synergy between the glycopeptide vancomycin with clarithromycin (Mukherjee et al., 2017).

Three novel hits showed encouraging synergy effects: Ramoplanin + tigecycline, vancomycin + tigecycline and teicoplanin + tigecycline inhibited growth at 5 + 0.8 μM, 2 + 1 μM and 3 + 1 μM, respectively (**Table 3**). Ramoplanin, vancomycin, and teicoplanin are all glycopeptides. Ramoplanin is not well absorbed and unstable in the bloodstream due to hydrolysis of the lactone bond (Farver et al., 2005). This makes ramoplanin unsuitable to repurpose for use in treatment of *M. abscessus* lung infections. As teicoplanin shows systemic exposure upon intravenous or intramuscular administration and has been reported to have a better safety and efficacy profile compared to vancomycin (Svetitsky et al., 2009), we characterized the activity of teicoplanin in combination with the glycylcycline tigecycline in more detail.

### Teicoplanin + Tigecycline Displays Activity Against Reference Strains Representing the Three Subspecies of the *M. abscessus* Complex

To determine whether the teicoplanin + tigecycline combination shows similar attractive potency across the three subspecies of the *M. abscessus* complex, we carried out the checkerboard titration assay to determine the FICI value of the combination against the reference strains *M. abscessus* subsp. *abscessus* ATCC 19977, *M. abscessus* subsp. *bolletii* CCUG 50184-T and *M. abscessus* subsp. *massiliense* CCUG 48898-T. The teicoplanin + tigecycline combination was synergistic against all three subspecies (**Table 4**). These results suggest that this novel combination is active across the phylogenetically divergent *M. abscessus* complex.

**Table 4:** Synergy concentrations and FICI values of teicoplanin + tigecycline combination against *M. abscessus* screening strain and three reference strains of the *M. abscessus* sub-species.

Conditions	Strains	MIC_90_ (μM)				FICI
			
		Teicoplanin		Tigecycline	
				
		Alone	Combined	Alone	Combined
(No pre-treatment)	*M. abscessus* Bamboo	25	3	8	1	0.27
	*M. abscessus* subsp. *abscessus*	10	2	6	2	0.42
	*M. abscessus* subsp. *bolletii*	10	2	8	2	0.39
	*M. abscessus* subsp. *massiliense*	14	2	7	2	0.36
Pre-treated with clarithromycin	*M. abscessus* subsp. *abscessus*	7	1.2	8.5	1.6	0.22


**‘Pre-treated with clarithromycin’ shows the respective data for a strain displaying induced macrolide resistance due to pre-treatment with a sub-inhibitory concentration of clarithromycin (see section “Materials and Methods”). Results shown are the mean of two replicates. Standard deviation was ±50% of shown values. Synergy is defined as FICI ≤ 0.5. Checkerboard assay was conducted with 7H9 broth*.*

### Teicoplanin + Tigecycline Retains Its Activity Against *M. abscessus* subsp. *abscessus* ATCC 19977 Cultures Displaying Induced Macrolide Resistance

The checkerboard titration assay was performed using *M. abscessus* subsp. *abscessus* ATCC 19977 cultures that had been exposed to a sub-inhibitory concentration of clarithromycin to induce macrolide resistance to determine whether the teicoplanin + tigecycline combination retains its activity under these conditions. The combination still exhibited synergy against the culture with induced macrolide resistance as seen by its FICI value of 0.22 (**Table 4**).

### Teicoplanin + Tigecycline Shows Potent Activity Against *M. abscessus* Clinical Isolates

The teicoplanin + tigecycline combination showed potent growth inhibition activity against the screening strain as well as the reference strains representing the three subspecies of *M. abscessus*. This suggests that most clinical *M. abscessus* strains may be susceptible to this combination. To provide evidence for a widespread susceptibility of *M. abscessus* to the teicoplanin + tigecycline combination, we tested its activity against a collection of clinical isolates covering various subspecies of *M. abscessus*, including clarithromycin resistant as well as clarithromycin sensitive strains. The combination displayed synergy against 70.4% of the isolates with FICI values ranging from 0.32 to 0.48 (**Table 5**). This result indicates that this combination is active against a large number of *M. abscessus* isolates.

**Table 5:** Synergy concentrations and FICI values of the teicoplanin + tigecycline combination against 14 clinical *M. abscessus* isolates.

Isolate code	*M. abscessus* sub-species	*erm41* sequevar	Clarithromycin susceptibility	MIC_90_ (μM)				FICI
					
				Teicoplanin		Tigecycline		
						
				Alone	Combined	Alone	Combined	
M199	*abscessus*	T28	Resistant	5	2	6	2	0.56
M337	*abscessus*	T28	Resistant	5	2	7	2	0.51
M404	*abscessus*	C28	Sensitive	6	2	4	1	0.53
M421	*abscessus*	T28	Resistant	17	3	4	0.6	0.37
M422	*abscessus*	T28	Resistant	6	1	6	1	0.44
M232	*bolletii*	T28	Resistant	21	5	18	2	0.39
M416	*bolletii*	N.D	Sensitive	4	2	5	1	0.63
M506	*bolletii*	C28	Sensitive	10	3	9	1	0.45
M111	*massiliense*	Deletion	Sensitive	10	2	10	2	0.48
M353	*massiliense*	Deletion	Sensitive	13	3	8	2	0.43
M357	*massiliense*	Deletion	Sensitive	12	2	8	1	0.33
M414	*massiliense*	Deletion	Sensitive	8	1	10	2	0.32
M444	*massiliense*	Deletion	Sensitive	7	3	6	1	0.48
M505	*massiliense*	Deletion	Sensitive	13	3	8	1	0.39


**Results shown are the mean of two replicates. Standard deviations were ±50% of shown values. Synergy is defined as FICI ≤ 0.5. Indifference is defined as 0.5 < FICI ≤ 4, and antagonism is defined as FICI > 4. N.D, not determined.**

### Teicoplanin + Tigecycline Is Not Bactericidal Against *M. abscessus* subsp. *abscessus* ATCC 19977

To determine whether the teicoplanin + tigecycline combination shows bactericidal activity against *M. abscessus*, cultures were treated with the drug combination and the effect on viability was determined by cfu enumeration as described in Section “Materials and Methods.” The teicoplanin + tigecycline combination showed no bactericidal activity.

## Discussion

A synergy screen of 180 dual antibiotic combinations against *M. abscessus* yielded a total of 11 hits. Six hits were obtained from combinations of β-lactams with β-lactamase inhibitors, and five hits from combinations of cell wall-targeting antibiotics with antibiotics that have intracellular targets.

From the analyses of combinations of β-lactams with β-lactamase inhibitors, the most striking observation is that all six hits involved a non-β-lactam-based β-lactamase inhibitor. 4 out of the 6 hits involved avibactam. This is consistent with previous reports showing that this inhibitor is effective against *M. abscessus*β-lactamases (Ehmann et al., 2012; Soroka et al., 2014; Dubee et al., 2015a; Kaushik et al., 2017). The remaining 2 hits involved the novel non-β-lactam-based β-lactamase inhibitor vaborbactam, which has not been previously studied for activity against *M. abscessus* β-lactamases. Activity of avibactam and now vaborbactam suggests that it may be worthwhile to characterize other types of non-β-lactam β-lactamase inhibitors like phosphonates, hydroxamates, or vanadate-catechol complexes, in combination with β-lactams for any potentiation effect of the combinations against *M. abscessus* (Bebrone et al., 2010).

It is to note that out of the three sub-classes of β-lactams we tested, avibactam appears not to improve the activity of cephalosporins, in contrast to a previous report describing potentiation between ceftaroline and avibactam against *M. abscessus* (Dubee et al., 2015b). A recent study by Kaushik et al. (2017) which focused on combinations of avibactam, sulbactam and tazobactam with carbapenems against *M. abscessus* reported 3 hits, with potentiation observed for combinations of avibactam with ertapenem, tebipenem, or panipenem (Kaushik et al., 2017). In this study, we could confirm potentiation for combinations of avibactam with tebipenem or panipenem, however, we did not observe any potentiation between avibactam and ertapenem. Another study found potentiation between clavulanate and meropenem, which we also did not observe (Kaushik et al., 2015). The reasons for these discrepancies remain to be determined (Fisher et al., 2005). One possible explanation may be the use of different *M. abscessus* strains. This study used the clinical isolate *M. abscessus* Bamboo in the initial screening, while other studies used other strains including *M. abscessus* subsp. *abscessus* ATCC19977. In comparison to avibactam, vaborbactam improved the activity of selected compounds only from the carbapenems but not from the penicillins.

Vaborbactam is a new β-lactamase inhibitor and is the first one to contain a cyclic boronic acid structure (Lomovskaya et al., 2017). Despite its difference in structure from avibactam, both β-lactamase inhibitors were able to potentiate the activity of the same two carbapenems, panipenem and tebipenem. Panipenem is an earlier carbapenem and the drug needs to be administered together with betamipron to block its deactivation by dehydropeptidase I (Papp-Wallace et al., 2011). Tebipenem is a more recently discovered carbapenem and is the first oral drug of this class (Papp-Wallace et al., 2011).

From the analyses of combinations of cell wall-targeting antibiotics with drugs that have intracellular targets, we obtained five novel hits, with the teicoplanin + tigecycline combination being most attractive. Teicoplanin + tigecycline combination displayed synergy at a similar range across reference strains representing the three subspecies of *M. abscessus* with growth inhibitory combination concentrations of 2–3 μM teicoplanin + 1–2 μM tigecycline. The combination also retained activity against most clinical isolates. A limitation of the combinations tested in this category is, that we only tested combinations of tigecycline with glycopeptides and not with other classes of cell wall-targeting antibiotics such as β-lactams, and this should be explored in future studies.

Teicoplanin is a glycopeptide and acts by interacting with the D-ala-D-ala terminal of the muramyl-pentapeptide which results in inhibition of the cell wall peptidoglycan synthesis (Parenti, 1986). The drug is reported to have good tissue and cellular penetration (Parenti, 1986). Teicoplanin was found to have lower adverse event rates compared to the glycopeptide vancomycin (Svetitsky et al., 2009). Tigecycline is a glycylcycline acting via inhibiting protein synthesis (Olson et al., 2006). Both teicoplanin and tigecycline are administered intravenously, which may limit their application. However, it is noteworthy that despite this limitation tigecycline is used to treat *M. abscessus* infections (Wallace et al., 2014). The exact molecular mechanism by which the synergistic combination of teicoplanin + tigecycline exerts its activity remains to be determined. Tigecycline may have limited ability to penetrate the bacterium to gain access to its intracellular target. One may speculate that with the administration of teicoplanin together with tigecycline, teicoplanin is able to ‘weaken’ the bacterial cell wall and allow greater penetration of tigecycline into the bacterium.

## Conclusion

This study has identified teicoplanin + tigecycline as a novel synergistic combination against *M. abscessus in vitro*. The drug pair can now be tested in *M. abscessus* animal models of infection and/or in patients.

## Author Contributions

DA, VD, and TD conceived the idea, developed the strategy, and wrote the manuscript. DA carried out the experiments. JT provided and characterized the clinical isolates.

## Conflict of Interest Statement

The authors declare that the research was conducted in the absence of any commercial or financial relationships that could be construed as a potential conflict of interest.
